# Effects of human articular cartilage constituents on simultaneous diffusion of cationic and nonionic contrast agents

**DOI:** 10.1002/jor.24824

**Published:** 2020-08-28

**Authors:** Abhisek Bhattarai, Janne T. A. Mäkelä, Behdad Pouran, Heikki Kröger, Harrie Weinans, Mark W. Grinstaff, Juha Töyräs, Mikael J. Turunen

**Affiliations:** ^1^ Department of Applied Physics University of Eastern Finland Kuopio Finland; ^2^ Diagnostic Imaging Center Kuopio University Hospital Kuopio Finland; ^3^ Department of Orthopaedics University Medical Center Utrecht Utrecht The Netherlands; ^4^ Department of Orthopedics, Traumatology and Hand Surgery Kuopio University Hospital Kuopio Finland; ^5^ Department of Biomechanical Engineering, Faculty of Mechanical, Maritime, and Materials Engineering Delft University of Technology (TU Delft) Delft The Netherlands; ^6^ Departments of Biomedical Engineering, Chemistry, and Medicine Boston University Boston Massachusetts; ^7^ School of Information Technology and Electrical Engineering The University of Queensland Brisbane Australia; ^8^ SIB Labs University of Eastern Finland Kuopio Finland

**Keywords:** collagen, contrast‐enhanced, proteoglycan, water

## Abstract

Contrast‐enhanced computed tomography is an emerging diagnostic technique for osteoarthritis. However, the effects of increased water content, as well as decreased collagen and proteoglycan concentrations due to cartilage degeneration, on the diffusion of cationic and nonionic agents, are not fully understood. We hypothesize that for a cationic agent, these variations increase the diffusion rate while decreasing partition, whereas, for a nonionic agent, these changes increase both the rate of diffusion and partition. Thus, we examine the diffusion of cationic and nonionic contrast agents within degraded tissue in time‐ and depth‐dependent manners. Osteochondral plugs (*N* = 15, *d* = 8 mm) were extracted from human cadaver knee joints, immersed in a mixture of cationic CA4+ and nonionic gadoteridol contrast agents, and imaged at multiple time‐points, using the dual‐contrast method. Water content, and collagen and proteoglycan concentrations were determined using lyophilization, infrared spectroscopy, and digital densitometry, respectively. Superficial to mid (0%‐60% depth) cartilage CA4+ partitions correlated with water content (*R* < −0.521, *P* < .05), whereas in deeper (40%‐100%) cartilage, CA4+ correlated only with proteoglycans (*R* > 0.671, *P* < .01). Gadoteridol partition correlated inversely with collagen concentration (0%‐100%, *R* < −0.514, *P* < .05). Cartilage degeneration substantially increased the time for CA4+ compared with healthy tissue (248 ± 171 vs 175 ± 95 minute) to reach the bone‐cartilage interface, whereas for gadoteridol the time (111 ± 63 vs 179 ± 163 minute) decreased. The work clarifies the diffusion mechanisms of two different contrast agents and presents depth and time‐dependent effects resulting from articular cartilage constituents. The results will inform the development of new contrast agents and optimal timing between agent administration and joint imaging.

## 1 INTRODUCTION

Articular cartilage is avascular, and its metabolic function is regulated via diffusion and convection of charged and uncharged solutes between the synovial fluid and the constituents of the cartilage extracellular matrix (ECM).^1^ Cartilage ECM is a heterogeneous structure, mainly consisting of interstitial water (60%‐85%), collagen fibrils (50%‐80% of dry content), and negatively charged proteoglycans (PGs; 20%‐30% of dry content).^2^, ^3^ Changes in the tissue composition alter the interstitial fluid flow ^2^, ^4^ and mechanical properties.^5^, ^6^, ^7^ The diffusion of a contrast agent inside the tissue, followed by subsequent contrast‐enhanced imaging, provides information on the health status of the cartilage tissue.^8^, ^9^, ^10^, ^11^, ^12^ For example, contrast‐enhanced computed tomography (CECT) is used to evaluate osteoarthritis (OA)‐related degeneration of cartilage and the associated alterations in the composition and morphology.^13^, ^14^, ^15^, ^16^

CECT diffusion studies of articular cartilage typically employ a single contrast agent.^11^, ^13^, ^14^, ^17^ In diffusion equilibrium, the partition of a nonionic agent follows the depth‐wise profile of the interstitial water content.^3^, ^15^ However, since OA‐related degeneration of cartilage affects all cartilage constituents, as well as the structure, sensitive quantification of cartilage health based on the partition of only a nonionic agent, is challenging. Anionic agents similarly suffer from low sensitivity, as they diffuse against the fixed negative charge that prevails inside healthy articular cartilage. In contrast, cationic contrast agents molecules are attracted into the tissue through electrostatic attraction and are used to directly quantify cartilage PG concentration.^12^, ^14^, ^18^

**Figure 1:**
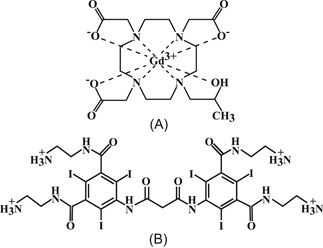
Molecular structure of (A) gadoteridol and (B) CA4+

Unhealthy articular cartilage possesses a disorganized collagen fibril network and increased permeability; thus facilitating agent diffusion.^19^, ^20^ However, the fixed charge density is concurrently reduced, because of the decrease in PG concentration, which slows down the diffusion of cationic agents. The combination of the two simultaneous and opposite effects complicates the interpretation of the acquired results, which in turn reflects the overall tissue health. To address this challenge, we recently introduced a dual‐contrast agent technique. In this technique, two CT‐based contrast agents (iodine I‐based cationic, CA4+)^21^ and gadolinium Gd‐based nonionic agent [gadoteridol]) are employed simultaneously (Figure 1) and the molar concentrations of the agents are quantified using a dual‐energy CT scan.^12^, ^22^, ^23^ The premise is that normalization of the cationic contrast agent partition with that of the nonionic contrast agent allows early diagnostics, as the changes in the tissue's steric hindrance are accounted for. The dual‐contrast method shows improved sensitivity and assessment of cartilage properties.^12^, ^22^, ^23^ However, questions still remain regarding the effects of the cartilage constituents and its hierarchical structure on the diffusion, for example, how the contrast agent flux in the superficial zone of cartilage differs from that in the deep cartilage, and how agent diffusion relates to the variation in the depth‐wise organization of the cartilage constituents?

In this study, we characterized the effects of the main cartilage constituent content, that is, PGs, collagen, and water, and their changes during OA‐related cartilage degradation, on the simultaneous diffusion of cationic and nonionic contrast agents. We evaluated the composition of the human articular cartilage samples via microscopy and spectroscopy and measured the diffusion of the contrast agents by dual‐contrast CECT.

## 2 MATERIALS AND METHODS

### 2.1 Sample extraction and microCT imaging

**Figure 2:**
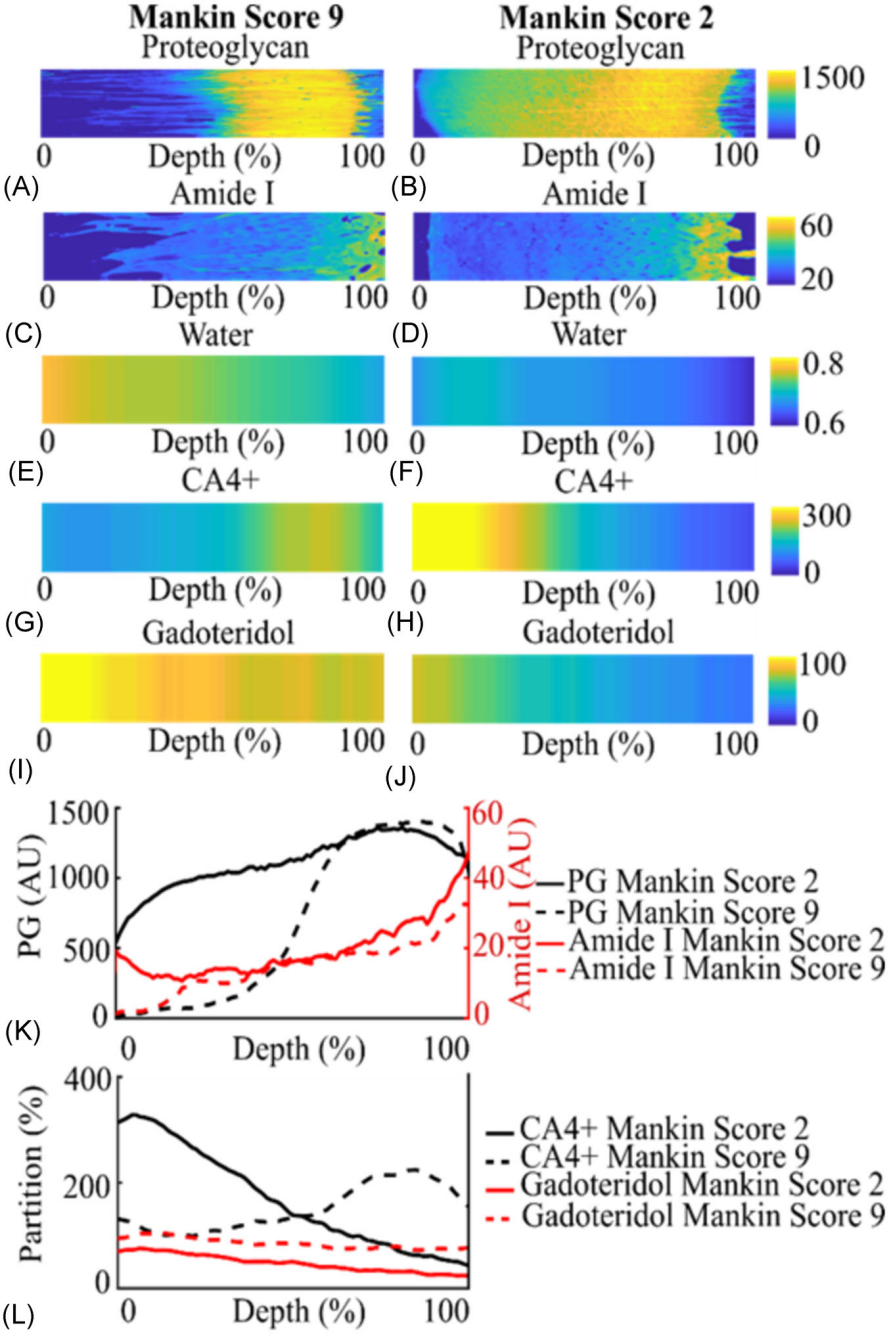
Depth‐wise proteoglycan (A, B, and K) concentration, amide I (C, D, and K) concentration, and water (E and F) content in the human articular cartilage samples. CA4+ (G, H, and L), and gadoteridol (I, J, and L) partitions in samples with Mankin scores of 9 and 2 after 10 hour of contrast agent diffusion (not in equilibrium). [Color figure can be viewed at wileyonlinelibrary.com]

Human osteochondral plugs (*N* = 15, *d* = 8 mm) were extracted from the proximal tibiae and distal femora of left and right knee joints of four cadavers (male 1: 68 years, male 2: 68 years, male 3: 69 years, and female 1: 79 years of age). The research committee of the North Savo Hospital District (Kuopio University Hospital, Finland) gave a favorable opinion (statement number: 134/2015 [58/2013]) for the sample collection. After the extraction, the plugs were halved to separately conduct diffusion experiments and reference measurements (Figure 2). For the CECT experiment, diffusion of the contrast agent mixture was allowed only through the articulating surface by sealing the edges using cyanoacrylate (Superglue Precision, Loctite, Düsseldorf, Germany). The plugs were immersed in a contrast agent bath (5 mL, osmolality: 297 mOsm/kg, 4°C) comprising of CA4+, which is a hydrochloride salt of 5,5′‐(malonylbis[azanediyl])bis(***N*** ^***1***^,***N*** ^***3***^‐bis(2‐aminoethyl)‐2,4,6‐triiodoisophthalamide) (molecular formula: C_27_H_36_C_l4_I_6_N_10_O_6_, *q* = +4, *M* = 1499 g/mol, 10 mgI/mL) and gadoteridol (molecular formula: C_17_H_29_GdN_4_O_7,_ Prohance, Bracco International B V, Amsterdam, The Netherlands, *q* = 0, *M* = 559 g/mol, 20 mgGd/mL), diluted in phosphate‐buffered saline (PBS). The estimated molecular length and width of CA4+ is 29 Å and 18 Å, respectively.^24^ The molecular size of gadoteridol was measured with a freely available open‐source web‐application to be ~11 Å long and ~6 Å wide (MolView, 2015).^25^ The osmolality of the contrast agent bath was selected to be similar to physiological saline, which is safe for clinical application.^26^ The bath was supplemented with following proteolytic inhibitors: 5 mM of ethylenediaminetetraacetic acid (VWR International, France), 5 mM of benzamidine hydrochloride hydrate (Sigma‐Aldrich Inc), and penicillin‐streptomycin‐amphotericin (antibiotic antimycotic solution, stabilized, Sigma‐Aldrich Inc, St. Louis, MO). Plugs were imaged in the air with a high‐resolution microCT scanner (Quantum FX, Perkin Elmer) using an isotropic voxel size of 40 × 40 × 40 µm and 20 × 20 mm field of view at two X‐ray energies (tube voltages of 90 kV and 50 kV). Similarly, after 10 minute, 30 minute, and 1, 2, 3, 4, 6, 10, 21, 32, 50, and 72 hour of the immersion the samples were removed from the bath and imaged. During the immersion in the contrast agent, the baths were constantly stirred and kept at a temperature of 4°C.

### 2.2 Image analysis

From the microCT images of the osteochondral plugs, the cartilage surface and the cartilage‐bone interface were defined manually using a segmentation software (Seg3D, version 2.4.0, The University of Utah, Salt Lake City, UT). Depth‐wise X‐ray attenuation inside a selected cartilage volume of interest (2800 × 2000 µm × cartilage thickness) was analyzed using Matlab (R2018b, The Mathworks Inc, Natick, MA). The depth‐wise concentration profiles of I and Gd‐based contrast agents within the cartilage were resolved from the X‐ray attenuation profiles (90 kV and 50 kV), based on the Beer‐Lambert law and Bragg's additive rule of mixtures.^12^, ^22^, ^27^, ^28^ Time‐dependent contrast agent diffusion curves were determined for 20% thick sections (0%‐20%, 20%‐40%, 40%‐60%, 60%‐80%, and 80%‐100% of cartilage depth) by fitting the following equation to the diffusion data $C=C_{\max}\times [1-\text{exp}(-t/T)],$ where *C*_max_ is the contrast agent concentration maximum, *t* is the diffusion time, and *T* is the time required for the contrast agent to reach 63.2% of the maximum concentration.^18^ The diffusion of the contrast agents was examined separately for five 20% thick cartilage sections with a partition threshold of 20%. This threshold was chosen to ensure sufficient temporal and spatial resolutions for determination of the contrast agent diffusion times.

### 2.3 Reference methods

Water content measurements were carried out on the osteochondral halves used in the diffusion experiments. The contrast agents were washed out by immersing the halves in PBS solution, supplemented with proteolytic inhibitors and penicillin‐streptomycin‐amphotericin for 5 days, while constantly stirred and refrigerated at a temperature of 4°C. The samples were then embedded (LAMB‐OCT, Thermo Fisher Scientific, Waltham, MA), fixed onto a frozen metallic sample holder, and placed inside a cryomicrotome (Leica CM3050 S, Leica Biosystems, Weltzar, Germany) chamber maintained at −21°C. To allow depth‐dependent characterization, 200 µm thick cartilage sections were cut along the transverse plane from the articulating surface until the cartilage‐bone interface, corresponding to an average of 11 slices per sample. The average thickness of the plugs was 2.35 ± 0.55 mm. The cut slices were freeze‐dried inside a lyophilizer chamber (Christ, Alpha 1‐2, B. Braun Biotech International, 37520 Osterode, Germany) for 48 hour by maintaining pressure 610.61 Pa. Each slice was weighed three times before and after the lyophilization and averaged. Depth‐wise water content was then obtained by subtracting the dry weight with the wet weight of the slice.

To determine the PG concentration distribution, 3‐µm thick sections were cut from the second half of the plug allocated for the reference measurements. The sections were stained with Safranin‐O, and quantitative digital densitometry (DD) measurements (Figure 2A,B) were conducted using a light microscope (Nikon Microphot‐FXA, Nikon Co, Japan) equipped with a monochromatic light source (*λ* = 420 ± 5 nm) and a 12‐bit CCD (ORCA‐ER, Hamamatsu Photonics K.K., Japan).^29^ Before the DD measurements, the system was calibrated using neutral density filters (Schott, Germany) with an OD range between 0 and 3. From the DD measurements, depth‐wise OD profiles from the cartilage surface to cartilage‐bone interface were calculated (Figure 2K).

Collagen concentration distribution was determined using Fourier Transform Infrared (FTIR) microspectroscopy system (Agilent Cary 670/620, Agilent Technologies Inc, Santa Clara, CA). For this, 3‐µm thick sections were prepared from an area adjacent to the sections prepared for Safranin‐O staining. Before the measurements, paraffin was removed, and the sections were moved onto Zinc‐Selenide windows. Similar regions of interest were selected from three sections per sample covering the full thickness of cartilage (Figure 2C‐D). The pixel size of 5.5 ×5.5 µm, spectral resolution 8 cm^−1^, and eight repeated scans were selected to measure the spatially resolved infrared spectra of the cartilage. The infrared light absorption spectrum in each pixel was collected within the wavelength range of 3800 to 750 cm^−1^ and, amide I concentration was measured from the peak area ranging from 1720 cm^−1^ to 1595 cm^−1^.^30^ The depth‐wise amide I concentration profiles were averaged from three sections per sample.

Histopathological Mankin score was assigned for the Safranin‐O stained cartilage sections by four independent observers.^31^ The grading (three sections per sample) is based on: (a) staining (0‐4), (b) tidemark integrity (0‐1), (c) abnormality in structure (0‐6), and (d) cellularity (0‐3). The Mankin score of the sections assigned by all the observers was finally averaged (Figure 2K).

### 2.4 Statistical analysis

**Figure 3:**
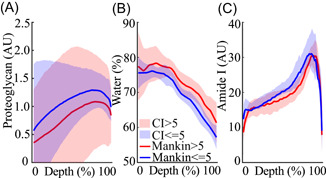
Depth‐wise profiles with confidence intervals (CIs) of (A) proteoglycan concentration, (B) water content, and (C) collagen (amide I) concentration in human articular cartilage samples with Mankin score ≤5 and Mankin score > 5. [Color figure can be viewed at wileyonlinelibrary.com]

To evaluate the effect of cartilage degeneration on contrast agent diffusion the samples were grouped based on the Mankin score (“more degenerated”: Mankin score > 5, *n* = 8, average score = 6.9 ± 1.1; “less degenerated”: Mankin score ≤ 5, *n* = 7, average score = 4.6 ± 1.3). Depth‐wise PG and collagen concentrations, and water content profiles were normalized to the length of 100 points and averaged (Figure 3). The association between the contrast agent partitions and the cartilage reference parameters was evaluated using Pearson's correlation. For all statistical tests, *P* < .05 was set as the limit of statistical significance. The significance of the difference in correlation coefficients between groups was tested with the Zou's method.^32^ Throughout this paper, the average descriptive values of the sample properties are presented as mean ± SD. All statistical analyses were conducted using SPSS (ver 23.0 SPSS Inc, IBM Company, Armonk, NY).

## 3 RESULTS

Histological analyses showed that PG and amide I concentrations predominantly increased while water content decreased as a function of cartilage depth (Figure 3).^30^, ^33^, ^34^, ^35^ The differences between the distributions of collagen (amide I) and PG concentration between more (Mankin score > 5) and less degenerated samples (Mankin score ≤ 5) were not statistically significant.

### 3.1 Diffusion as a function of cartilage depth

**Figure 4:**
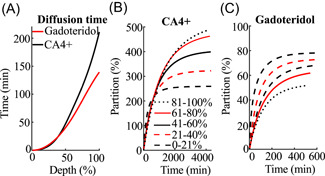
(A) The time required for the contrast agents partition to reach 20% of the bath concentration in each cartilage section. (B) CA4+ and, (C) gadoteridol partitions as a function of diffusion time at different 20% thick cartilage depths (sections). The initiation of diffusion in each section is assumed to begin when the contrast agent partition in the section reaches 20% of the contrast agent bath concentration. [Color figure can be viewed at wileyonlinelibrary.com]

The rate of diffusion was similar for CA4+ and gadoteridol until the agents reached 40% of the cartilage depth (Figure 4A). The average time (all the samples) for gadoteridol to reach the cartilage‐bone interface was 141 ± 83 minute and for the CA4+ it was 216 ± 165 minute. This difference was statistically significant (*P* < .01) for the more degenerated samples where the time for gadoteridol was 111 ± 63 minute and for CA4+ 248 ± 171 minute. For the less degenerated samples, the times were 179 ± 163 minute and 175 ± 95 minute, respectively.

### 3.2 Effects of cartilage constituents to the diffusion

**Figure 5:**
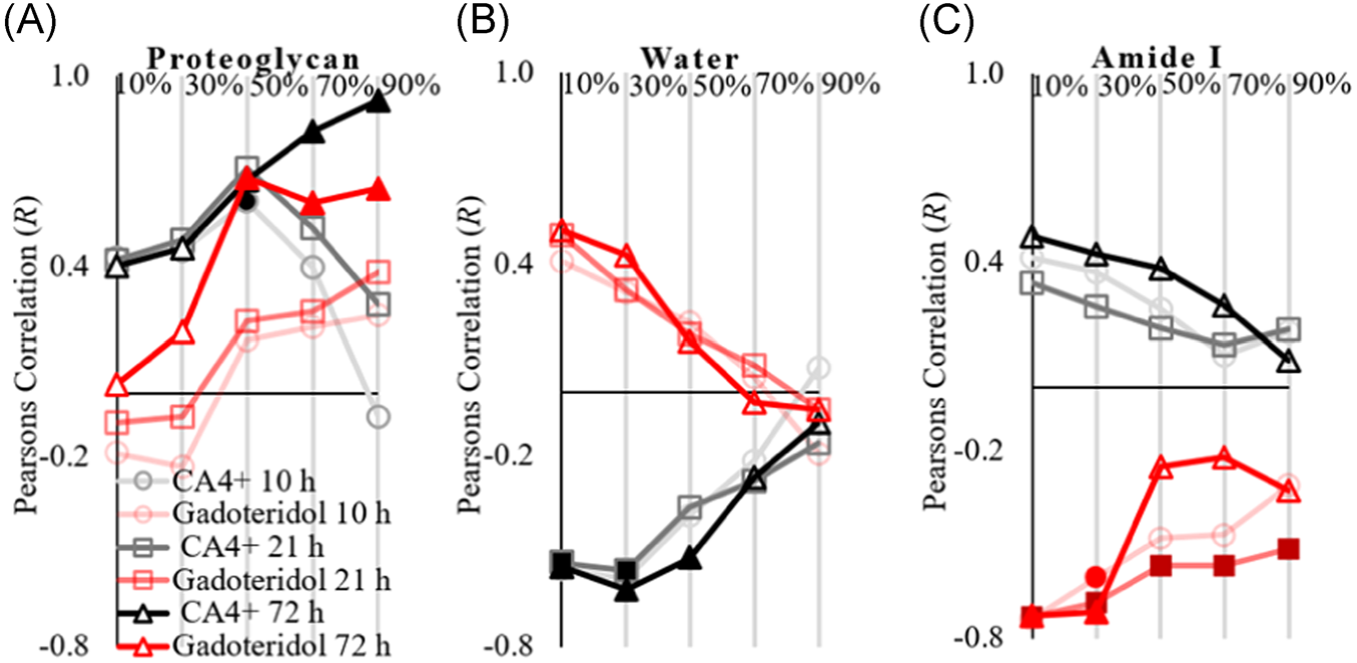
Pearson's correlation coefficients between maximum contrast agent concentration (*C*_CA4+ max_ and *C*_Gd max_) and cartilage (A) proteoglycan concentration, (B) water content, and (C) collagen (amide I) concentration after 10, 21, and 72 hour of diffusion. Solid markers indicate a statistically significant correlation (*P* < .05) [Color figure can be viewed at wileyonlinelibrary.com]

**Figure 6:**
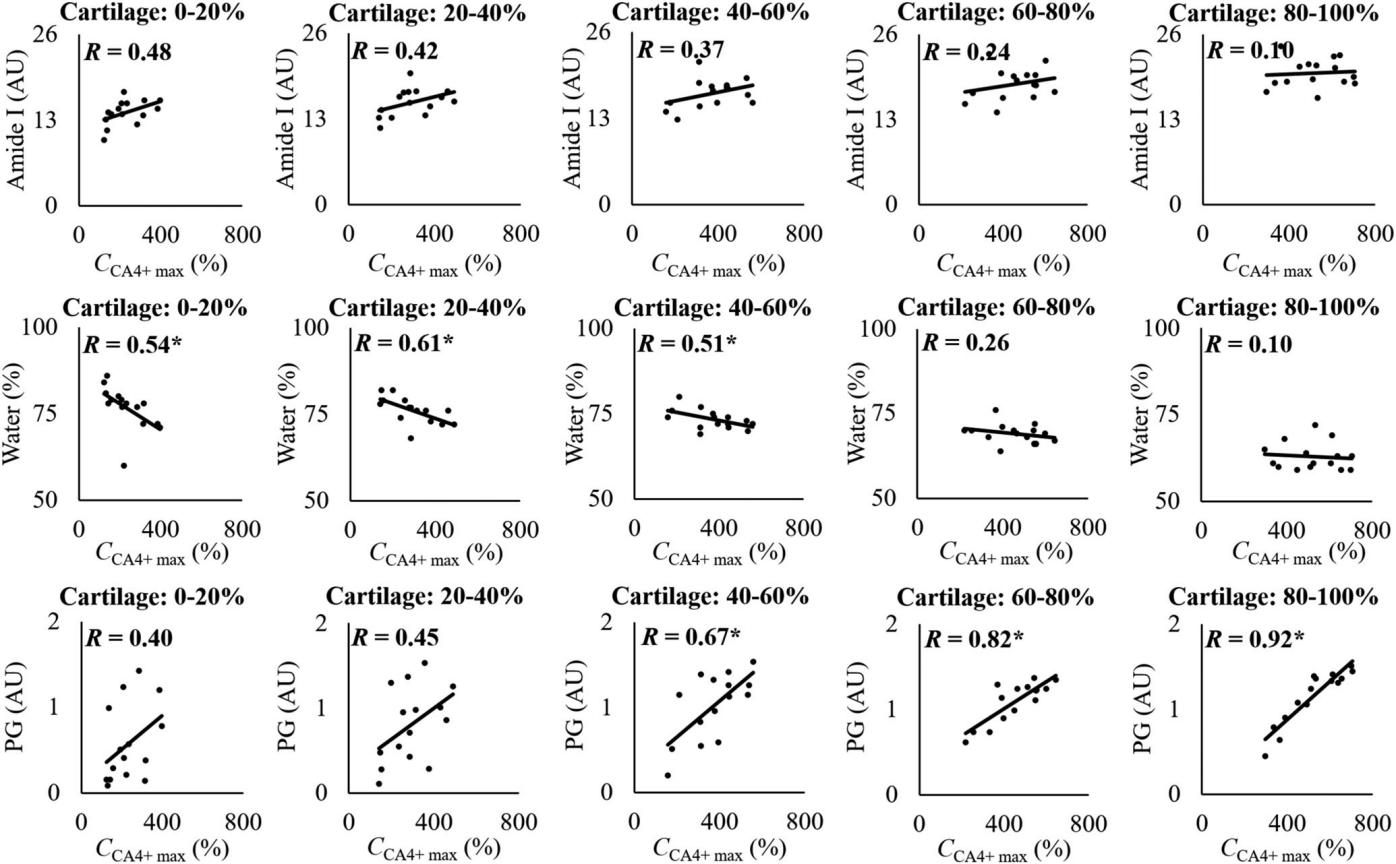
Scatterplots showing linear Pearson's correlations (*R*) between CA4+ maximum concentration at 72 hour (the time point closest to the diffusion equilibrium) and collagen (amide I), water, and proteoglycan (PG) concentrations at different 20% thick cartilage sections. Statistical significance is indicated with * when *P* < .05

**Figure 7:**
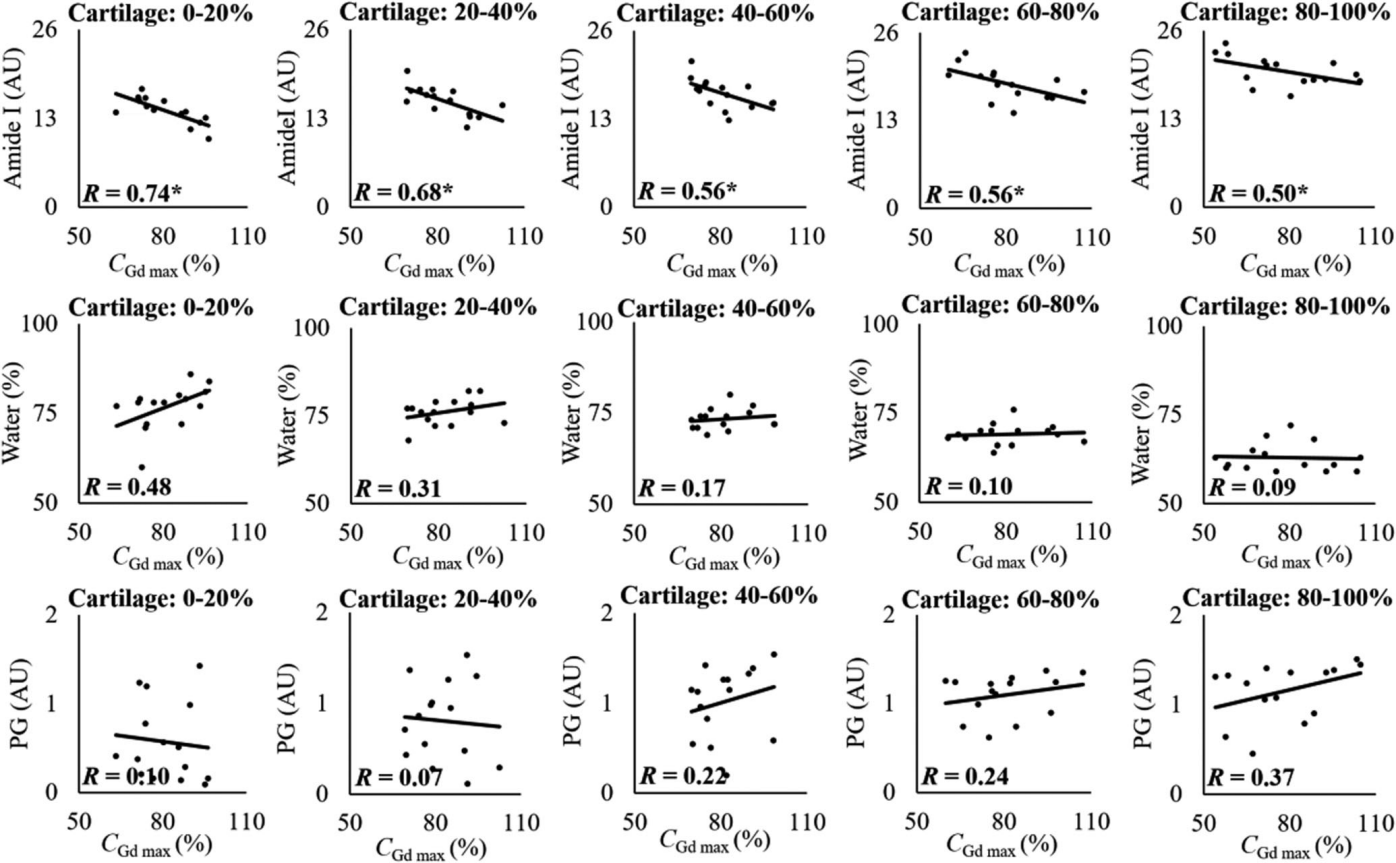
Scatterplots showing linear Pearson's correlations (*R*) between gadoteridol maximum concentration at 21 hour (the time point closest to the diffusion equilibrium) and collagen (amide I), water, and proteoglycan (PG) contents at different 20% thick cartilage sections. Statistical significance is indicated with * when *P* < .05

The correlation between the cartilage constituents and the contrast agent partitions were studied at three time‐points: 10, 21, and 72 hour. CA4+ concentration maximum (*C*_CA4+ max_, 72 hour) correlated significantly with the PG concentration (*R* > 0.671, *P* < .01) in the deeper cartilage (40%‐100% of cartilage thickness) (Figures 5A and 6). At 72 hour, we observed a significant inverse correlation (*R* < −0.521, *P* < .05) with water content from the surface until 60% of cartilage depth (Figures 5B and 6). The maximum gadoteridol concentration (*C*_Gd max_) correlated inversely with collagen concentration (*R* < −0.514, *P* < .05) at 21 hour of diffusion throughout the cartilage thickness (Figures 5C and 7). At the 72 hour time‐point, *C*_Gd max_ correlated inversely with the collagen concentration (*R* < −0.705, *P* < .01) at the superficial 40% of cartilage depth and correlated positively (*R* > 0.567, *P* < .01) with PG concentration from 40% depth until the cartilage‐bone interface (Figures 5A and 7).

## 4 DISCUSSION

In this study, we evaluate the effects of human articular cartilage constituents and structure on the simultaneous diffusion of cationic and nonionic contrast agents. By correlating the depth‐wise composition of cartilage with the maximum contrast agent partitions, we show that the CA4+ partition in the superficial (0%‐20%) and initial middle zone (20‐40) is governed by the PG concentration and to a greater extent by the tissue water content (*R* = 0.4 vs *R* = 0.54* and *R* = 0.45 vs *R* = 0.61*, respectively, *P < .05). However, the differences in the correlations are not significant (Zou's method).^32^ In the later middle (40%‐60%) and deep (60‐80 and 80%‐100%) zones, the CA4+ partition strongly and significantly correlates with the PG concentration (*R* = 0.67*, *R* = 0.82*, and *R* = 0.92*, respectively, *P < .05). This finding is consistent with the lower concentration of PG in the superficial/middle zones and the PG gradient present in cartilage.^36^, ^37^ In addition, contrary to the general perception,^15^, ^36^ the gadoteridol partition did not correlate with the water content. Instead, we observed a strong inverse relationship with the collagen concentration.

PG concentration governs CA4+ diffusion via the electrostatic attraction induced by the fixed negative charge.^38^ A positive correlation between *C*_CA4+ max_ and the PG concentration exists in the middle to deep cartilage (ie, 40% depth to the calcified cartilage layer) (Figures 5A and 6). However, based on the current results, the PGs alone does not govern the diffusion of CA4+ in the superficial and middle zones (ie, from the articulating surface to ~40% of the cartilage depth). The *C*_CA4+ max_ inversely correlates with cartilage water content. This might be due to the loss of PGs or an increase in the water content in the superficial and middle zones, resulting from the loss of collagen integrity.^2^ As expected, the collagen concentration had no direct effect on *C*_CA4+ max_ (Figures 5C and 7). Even though the depth‐wise gadoteridol partition resembles the water distribution in cartilage (Figure 3B), the expected association between the water content and *C*_Gd max_ ^3^, ^39^ are not statistically significant. Instead, *C*_Gd max_ inversely correlates with the collagen concentration. This relation is a result of the collagen being the main solid constituent of the cartilage. In degenerated cartilage, the resulting collagen fibrillation allows more free fluid flow, that is, increased permeability and allowing swifter diffusion of contrast agents.^20^, ^40^ However, an inverse correlation exists between the water content and collagen concentration (*R* = −0.62, *P* < .05; Figure S1).

Structural degradation of cartilage, that is, collagen fibrillation and an increase in water content are important factors affecting the diffusion of the contrast agents (Figure 2). Our results show that the diffusion of the contrast agents is nonuniform throughout the thickness of the cartilage (Figure 4). The time required to reach 20% partition in the deep cartilage is longer for CA4+ than for gadoteridol (Figure 4A), and the time increases with advancing cartilage degeneration (ie, increased Mankin score). We surmise that this result is due to: (a) the larger molecule size of CA4+ (29 × 18 Å) compared with gadoteridol (11 × 6 Å); (b) degradation related decrease in PG concentration, reducing the electrostatic attraction, which is especially pronounced in the superficial and middle regions (Figure 3); and/or (c) the multivalent electrostatic interactions between CA4+ and PGs as it traverses the tissue, slowing the diffusion. All of the aforementioned factors result in increased time for the agent to reach deeper into the cartilage‐bone interface. Previous studies reported a decrease in permeability towards the deep cartilage, due to the gradual increase in PG concentration, and similar findings are reported herein (Figures 3A and 3C).^41^, ^42^ As revealed in the present study, in the more degenerated samples the time for the cationic agent to reach the cartilage‐bone interface is twice that of the nonionic agent, while no difference is seen with the less degenerated samples. These results add to the literature and further demonstrate that OA‐related degradation of cartilage and associated compositional variations affect the contrast agent's partitions and their diffusion rates.^43^, ^44^

The diffusion of cationic contrast agent (CA4+) in cartilage is governed by negatively charged PGs, tissue permeability, and water content. There were no significant correlations between amide I concentration and CA4+ partition in any diffusion time point or cartilage depth, and the correlations are similar between the diffusion time points (10, 21, and 72 hour) (Figure 5C). Gadoteridol reaches diffusion equilibrium between the 21 and 32 hour measurement time points (1745 minute). At this time‐point, no correlation exists between the gadoteridol partition and the PG concentration. However, at the 72 hour diffusion time point, the gadoteridol partition strongly correlates with the PG concentration, whereas the correlation with collagen (amide I) concentration observed in the 21 hour time point is not present in the mid to deep cartilage sections. High diffusion flux of CA4+ has been suggested to cause drag influencing diffusion of gadoteridol.^45^, ^46^ However, current data and experiments are not sufficient to state whether the high uptake of CA4+ in deep cartilage influenced gadoteridol diffusion and decrease in correlation between amide I concentration and gadoteridol partition at 72 hour time point. Authors suspect the high partition and diffusion flux of CA4+ affected gadoteridol partition at 72 hour diffusion time point. Hence, we presented the correlation between gadoteridol partition and cartilage constituent content earlier, that is, at 21 hour diffusion time point (when gadoteridol diffusion was near equilibrium).

There are some limitations associated with the current study. The diffusion experiments and the reference (histological and spectroscopic) measurements were performed on the adjacent regions of the halved plugs. This might add error to the comparison between the diffusion properties and the reference data. However, since the regions were adjoining, we assume the state of samples to be relatively homogeneous across the halved plugs. The diffusion in cartilage was examined in a time‐ and depth‐dependent manner, which required intact cartilage. The samples could not be sliced for water content measurement prior to the diffusion experiment. Even after washing out the contrast agents from the sample for 120 hours remnants of CA4+ might have persisted, adding to the weight of the slices. However, any remaining contrast agent would also stay attached during and after lyophilization, adding only a minimal error to the determined water content. The FTIR measurements provided the depth‐dependent concentration of collagen (amide I) content in cartilage. Fibrillation and alteration in collagen fiber orientation precede the loss in collagen.^47^ The present samples were mostly arthritic (average Mankin score = 5.6) with eroded superficial zones (Figure 3C), which affects contrast agent diffusion. The information on the collagen fibril organization would have added to the interpretation contrast agents' diffusion properties, and lack of this information is acknowledged as a limitation of this study. The diffusion of the contrast agents was examined separately for five 20% thick cartilage sections. The concentration of contrast agents in every section depends on the concentration of the preceding cartilage section and the values are related to the equilibrium concentration. Thus, the extraction of diffusion coefficients will be a premise of future study requiring finite element modeling.^48^

To conclude, the diffusion of cationic contrast agents depends not only on the PG concentration but also on the water content, especially in the superficial and middle zones of cartilage. The diffusion of nonionic agents inversely relates to cartilage collagen concentration. The degenerative state of the cartilage governs contrast agent's diffusion rates; with cartilage degeneration, the diffusion rates of nonionic and cationic contrast agents increase and decrease, respectively. The results presented in this study increase the knowledge base and understanding of how the contrast agent diffusion and the resulting partitions depend on the composition and OA‐related degradation of the articular cartilage. Furthermore, the present results will inform the timing between the contrast agent administration and the tomographic image acquisition.

## CONFLICT OF INTERESTS

The authors declare that there are no conflict of interests.

## AUTHOR CONTRIBUTIONS

AB, JTAM, JT, and MJT designed the study. HK applied for ethical approval to obtain the cartilage samples. AB and BP performed the contrast agent diffusion experiment in the laboratory of HW. AB conducted water content measurements and digital densitometry analyses. AB and MJT conducted collagen content measurements and analyses. CA4+ was prepared in the laboratory of MG. AB, JTAM, JT, and MJT were involved in data analysis and interpretation of the results. AB drafted the manuscript, and all coauthors contributed to the critical revision of the manuscript. All authors have read and approved the final version of the submitted manuscript.

## Supporting information

Supporting information
