# Supplementary material for: Effects of human articular cartilage constituents on simultaneous diffusion of cationic and nonionic contrast agents
Source: J Orthop Res. 2020 Aug 28;39(4):771–9. doi: 10.1002/jor.24824 (PMC8048551; doi:10.1002/jor.24824)
Supplement: Supplementary file 1 — Supporting information [file JOR-39-771-s001.docx]

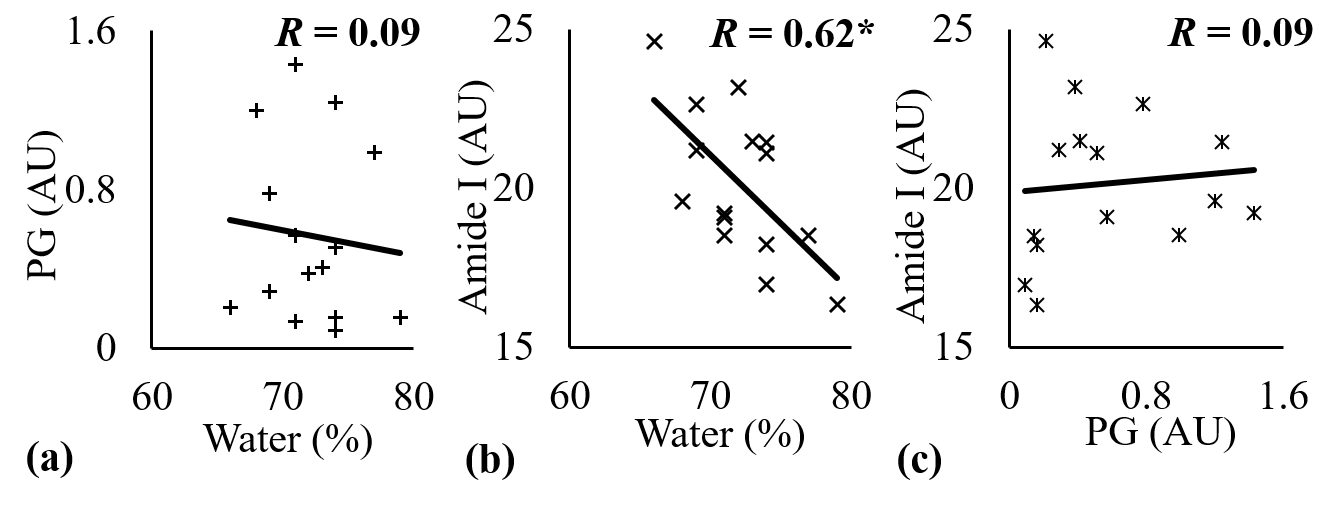
**Supplementary figure**

Fig. S-1. Scatterplots showing linear correlations between cartilage (a) proteoglycan (PG) and water, (b) collagen (amide I) and water, and (c) collagen and PG concentrations. Pearson correlation coefficients (R) are indicated with statistical significance * p < 0.05.
